# 2-Methyl­imidazolium picrate

**DOI:** 10.1107/S1600536810053390

**Published:** 2010-12-24

**Authors:** Grzegorz Dutkiewicz, S. Samshuddin, B. Narayana, H. S. Yathirajan, Maciej Kubicki

**Affiliations:** a Department of Chemistry, Adam Mickiewicz University, Grunwaldzka 6, 60-780 Poznań, Poland; b Department of Studies in Chemistry, Mangalore University, Mangalagangotri 574 199, India; c Department of Studies in Chemistry, University of Mysore, Manasagangotri, Mysore 570 006, India

## Abstract

In both ionic components of the title salt, C_4_H_7_N_2_^+^·C_6_H_2_N_3_O_7_^−^, the rings are approximately planar; the maximum deviation from the mean plane is an order of magnitude larger in the picrate ring [0.0289 (10) Å] than in the imidazolium ring [0.0028 (10) Å. The nitro groups are twisted with respect to the six-atom ring plane; the NO_2_ groups next to the oxide O atom, at the 2- and 6-positions, are twisted more [by 53.59 (9) and 18.46 (12)°] than the NO_2_ group at the 4-postition, for which the twist angle is 7.28 (16)°. In the crystal, N—H⋯O hydrogen bonds, in which the hydroxyl O atom acts as a double acceptor and one of the O atoms from a nitro group acts as an additional acceptor, connect mol­ecules into chains along the *c*-axis direction. Relatively short C—H⋯O contacts and π–π inter­actions between symmetry-related six-membered rings [centroid–centroid distances = 3.5938 (10) and 3.6223 (10) Å] also occur.

## Related literature

For the crystal structure of imidazolium picrate, see: Soriano-García *et al.* (1990 ▶). For the structures of picrates of some other imidazole derivatives, see, for example: Nardelli *et al.* (1987 ▶); Du & Zhao (2003 ▶); MacDonald *et al.* (2005 ▶); Pi *et al.* (2009 ▶).
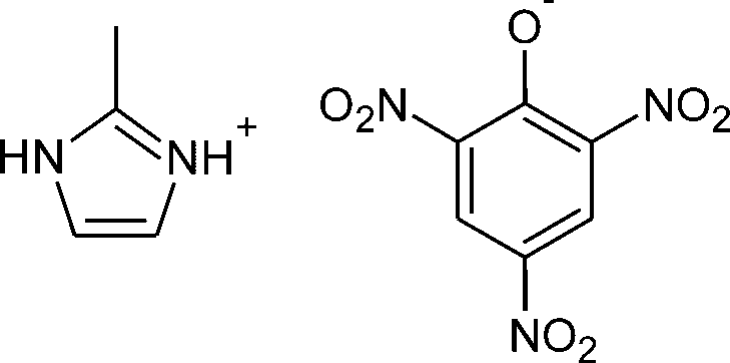


## Experimental

### 

#### Crystal data

C_4_H_7_N_2_^+^·C_6_H_2_N_3_O_7_^−^
*M* *_r_* = 311.22
Monoclinic, 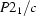

*a* = 7.0983 (9) Å
*b* = 21.644 (2) Å
*c* = 8.1583 (9) Å
β = 100.327 (12)°
*V* = 1233.1 (2) Å^3^
*Z* = 4
Mo *K*α radiation
μ = 0.15 mm^−1^
*T* = 295 K
0.3 × 0.2 × 0.2 mm

#### Data collection

Oxford Diffraction Xcalibur Eos diffractometer
Absorption correction: multi-scan (*CrysAlis PRO*; Oxford Diffraction, 2009 ▶) *T*_min_ = 0.964, *T*_max_ = 1.000
4778 measured reflections
2483 independent reflections
1792 reflections with *I* > 2σ(*I*)
*R*_int_ = 0.014

#### Refinement

*R*[*F* ^2^ > 2σ(*F* ^2^)] = 0.039
*wR*(*F* ^2^) = 0.101
*S* = 1.03
2483 reflections
235 parameters
All H-atom parameters refined
Δρ_max_ = 0.30 e Å^−3^
Δρ_min_ = −0.27 e Å^−3^

### 

Data collection: *CrysAlis PRO* (Oxford Diffraction, 2009 ▶); cell refinement: *CrysAlis PRO*; data reduction: *CrysAlis PRO*; program(s) used to solve structure: *SIR92* (Altomare *et al.*, 1993 ▶); program(s) used to refine structure: *SHELXL97* (Sheldrick, 2008 ▶); molecular graphics: *Stereochemical Workstation Operation Manual.* (Siemens, 1989 ▶); software used to prepare material for publication: *SHELXL97*.

## Supplementary Material

Crystal structure: contains datablocks I, global. DOI: 10.1107/S1600536810053390/dn2638sup1.cif

Structure factors: contains datablocks I. DOI: 10.1107/S1600536810053390/dn2638Isup2.hkl

Additional supplementary materials:  crystallographic information; 3D view; checkCIF report

## Figures and Tables

**Table 1:** Hydrogen-bond geometry (Å, °)

*D*—H⋯*A*	*D*—H	H⋯*A*	*D*⋯*A*	*D*—H⋯*A*
C5—H5⋯O21^i^	0.911 (19)	2.478 (19)	3.378 (2)	169.4 (16)
N11—H11⋯O1	0.89 (2)	1.95 (2)	2.8357 (19)	172.9 (19)
N13—H13⋯O1^ii^	0.86 (2)	2.09 (2)	2.819 (2)	143 (2)
N13—H13⋯O22^ii^	0.86 (2)	2.14 (2)	2.782 (2)	131.3 (19)

Symmetry codes: (i) 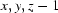; (ii) 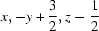.

**Fig. 1.**
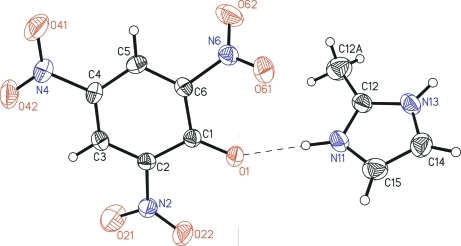
Anisotropic ellipsoid representation of the compound I together with atom labelling scheme. The ellipsoids are drawn at the 50% probability level, hydrogen atoms are depicted as spheres with arbitrary radii; hydrogen bond is shown as dashed line/

**Fig. 2.**
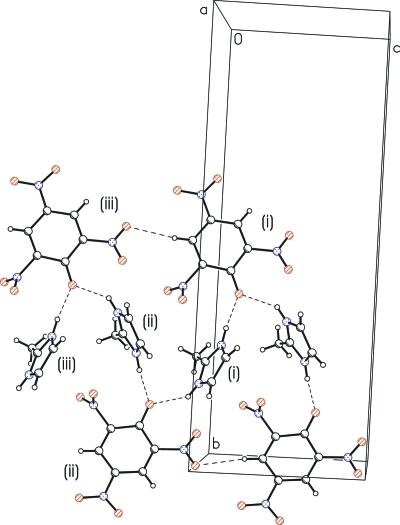
The layer of molecules of I as seen approximately along x direction; hydrogen bonds are shown as dashed lines. Syzmmetry codes: (i) x,3/2 - y,-1/2 + z; (ii) x,y,-1 + z; (iii) x,3/2 - y,-3/2 + z.

**Fig. 3.**
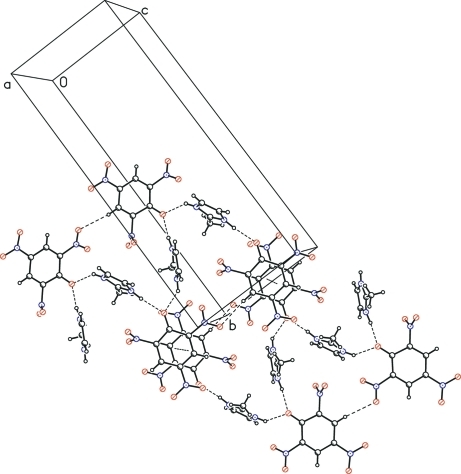
Two layers related by the center of symmetry; hydrogen bonds and π–π contacts are shown as dashed lines.

## supplementary crystallographic information

### Comment

The imidazole nucleus appears in a number of naturally occurring products like amino acid histidine and purines which comprise many of the most important bases in nucleic acids. Imidazole derivatives deal with a broad spectrum of pharmacological activities. The crystal structures of some imidazolium picrates have been reported, for instance imidazolium picrate itself (Soriano-García *et al.* (1990); also two solvates (hydrate and ethanolate) of 2-aminohistamine dipicrate (Nardelli *et al.*,1987), 4-hydroxymethylimidazolium picrate (Du & Zhao, 2003), two polymorphs of betaine bis(diimidazolium) dipicrate (MacDonald *et al.*, 2005) and 3-benzyl-1-methyl-imidazolium picrate (Pi *et al.*, 2009).

The title compound, 2-methylimidazolium picrate (2-methyl-1*H*-imidazol-3-ium 2,4,6-trinitrophenolate, Scheme 1) crystallizes with two ionic components, as proved by successful location and refinement of hydrogen atoms at both imiadzole nitrogen atoms as well as by the bond length pattern. Both cyclic fragments are in a good approximation planar. As might be expected, the deviations from the least-squares plane are by an order of magnitude smaller in the imidazolium ring (maximum 0.0028 (10) Å) than in a six-atom ring of picrate anion (0.0289 (10) Å). The two rings make a dihedral angle of 60.28 (7)°. The nitro groups are twisted with respect to the ring plane; the dihedral angles are larger for the substituents *ortho*- with respect to the C=O^-^ group (18.46 (12)° and 53.59 (9)°) than for the *para*-group, which is 7.28 (16)°.

The ionic fragments are conneced by relatively short N—H···O hydrogen bonds. One of N—H groups acts as a donor in almost linear hydrogen bond, while the other is involved in the bifurcated N—H···O bonds, in which O1 atom and one of nitro group O atoms act as acceptors. These two bonds are of similar lengths and therefore they deviate significantly from linearity. These hydrogen bonds, together with relatively short C—H···O contacts connect the cations and anions into a layer in which one can find the C^1^_2_(6) chains, created only by N—H···O hydrogen bonds and *R*^4^_5_(21) rings, in which all contacts are involved (Fig. 2).

The layers related by centers of symmetry are additionally connected by quite short π–π stacking interactions between six-membered rings. The distances between the centers of consecutive rings in a stack are 3.594Å and 3.621Å, but if the parallel shift is taken into account the distances between the planes are 3.48Å and 3.27Å (Fig. 3). It should be noted, however, that the parallel shift is in these cases 1.00Å and 1.50Å, respectively, which might suggest weak π–π stacking in the first case but edge-to-edge kind of interaction in the second.

### Experimental

2-Methyl imidazole (0.82 g, 0.01 mol) was dissolved in 25 ml of ethanol. Picric acid (2.29 g, 0.01 mol) was dissolved in 15 ml of water. Both the solutions were mixed and to this, 5 ml of 5*M* HCl was added and stirred for few minutes. The formed complex (I) was filtered and dried. Good quality crystals were grown from ethanol solution by slow evaporation (m. p.: 483 K). Composition: Found (Calculated): C: 38.50 (38.59); H: 2.88 (2.91); N: 22.45 (22.50).

### Refinement

Hydrogen atoms were located in difference Fourier maps and isotropically refined.

### Crystal data

**Table:**

C_4_H_7_N_2_^+^·C_6_H_2_N_3_O_7_^−^	*F*(000) = 640
*M**_r_* = 311.22	*D*_x_ = 1.676 Mg m^−^^3^
Monoclinic, *P*2_1_/*c*	Mo *K*α radiation, λ = 0.71073 Å
Hall symbol: -P 2ybc	Cell parameters from 2592 reflections
*a* = 7.0983 (9) Å	θ = 2.9–28.2°
*b* = 21.644 (2) Å	µ = 0.15 mm^−^^1^
*c* = 8.1583 (9) Å	*T* = 295 K
β = 100.327 (12)°	Block, yellow
*V* = 1233.1 (2) Å^3^	0.3 × 0.2 × 0.2 mm
*Z* = 4	

### Data collection

**Table:**

Oxford Diffraction Xcalibur Eos diffractometer	2483 independent reflections
Radiation source: Enhance (Mo) X-ray Source	1792 reflections with *I* > 2σ(*I*)
graphite	*R*_int_ = 0.014
Detector resolution: 16.1544 pixels mm^-1^	θ_max_ = 28.2°, θ_min_ = 2.9°
ω scans	*h* = −8→9
Absorption correction: multi-scan (*CrysAlis PRO*; Oxford Diffraction, 2009)	*k* = −15→28
*T*_min_ = 0.964, *T*_max_ = 1.000	*l* = −10→10
4778 measured reflections	

### Refinement

**Table:**

Refinement on *F*^2^	Primary atom site location: structure-invariant direct methods
Least-squares matrix: full	Secondary atom site location: difference Fourier map
*R*[*F*^2^ > 2σ(*F*^2^)] = 0.039	Hydrogen site location: inferred from neighbouring sites
*wR*(*F*^2^) = 0.101	All H-atom parameters refined
*S* = 1.03	*w* = 1/[σ^2^(*F*_o_^2^) + (0.060*P*)^2^] where *P* = (*F*_o_^2^ + 2*F*_c_^2^)/3
2483 reflections	(Δ/σ)_max_ = 0.001
235 parameters	Δρ_max_ = 0.30 e Å^−^^3^
0 restraints	Δρ_min_ = −0.27 e Å^−^^3^

### Special details

**Table:**

Geometry. All e.s.d.'s (except the e.s.d. in the dihedral angle between two l.s. planes) are estimated using the full covariance matrix. The cell e.s.d.'s are taken into account individually in the estimation of e.s.d.'s in distances, angles and torsion angles; correlations between e.s.d.'s in cell parameters are only used when they are defined by crystal symmetry. An approximate (isotropic) treatment of cell e.s.d.'s is used for estimating e.s.d.'s involving l.s. planes.
Refinement. Refinement of *F*^2^ against ALL reflections. The weighted *R*-factor *wR* and goodness of fit *S* are based on *F*^2^, conventional *R*-factors *R* are based on *F*, with *F* set to zero for negative *F*^2^. The threshold expression of *F*^2^ > σ(*F*^2^) is used only for calculating *R*-factors(gt) *etc*. and is not relevant to the choice of reflections for refinement. *R*-factors based on *F*^2^ are statistically about twice as large as those based on *F*, and *R*- factors based on ALL data will be even larger.

### Fractional atomic coordinates and isotropic or equivalent isotropic displacement parameters (Å^2^)

**Table:**

	*x*	*y*	*z*	*U*_iso_*/*U*_eq_	
C1	0.3265 (2)	0.93118 (8)	0.60988 (19)	0.0241 (4)	
O1	0.38378 (17)	0.88003 (5)	0.67241 (14)	0.0347 (3)	
C2	0.2787 (2)	0.98440 (7)	0.70037 (19)	0.0253 (4)	
N2	0.2874 (2)	0.98159 (7)	0.88019 (17)	0.0358 (4)	
O21	0.2016 (3)	1.02077 (8)	0.94412 (17)	0.0716 (5)	
O22	0.3770 (3)	0.94120 (7)	0.96128 (17)	0.0656 (5)	
C3	0.2171 (2)	1.03981 (8)	0.6272 (2)	0.0266 (4)	
H3	0.189 (2)	1.0723 (8)	0.691 (2)	0.028 (5)*	
C4	0.1956 (2)	1.04630 (8)	0.4569 (2)	0.0267 (4)	
N4	0.1304 (2)	1.10493 (7)	0.38329 (19)	0.0348 (4)	
O41	0.1247 (2)	1.11171 (7)	0.23347 (17)	0.0615 (5)	
O42	0.08086 (19)	1.14506 (6)	0.47167 (17)	0.0479 (4)	
C5	0.2260 (2)	0.99677 (8)	0.3565 (2)	0.0272 (4)	
H5	0.205 (3)	0.9999 (9)	0.243 (2)	0.041 (5)*	
C6	0.2876 (2)	0.94228 (7)	0.43176 (19)	0.0248 (4)	
N6	0.3054 (2)	0.89003 (7)	0.32303 (17)	0.0310 (3)	
O61	0.45451 (19)	0.86075 (6)	0.34477 (16)	0.0436 (4)	
O62	0.1676 (2)	0.87781 (7)	0.21488 (17)	0.0537 (4)	
N11	0.5304 (2)	0.76346 (7)	0.59823 (18)	0.0353 (4)	
H11	0.491 (3)	0.8016 (10)	0.617 (3)	0.054 (6)*	
C12	0.4264 (2)	0.72364 (8)	0.49579 (19)	0.0310 (4)	
C12A	0.2238 (3)	0.73045 (13)	0.4178 (3)	0.0484 (5)	
H12A	0.163 (5)	0.6936 (15)	0.401 (4)	0.129 (13)*	
H12B	0.155 (4)	0.7574 (13)	0.480 (3)	0.094 (9)*	
H12C	0.213 (4)	0.7461 (14)	0.318 (4)	0.111 (11)*	
N13	0.5382 (2)	0.67629 (7)	0.47868 (18)	0.0341 (4)	
H13	0.506 (3)	0.6450 (11)	0.416 (3)	0.056 (7)*	
C14	0.7165 (3)	0.68530 (9)	0.5723 (2)	0.0377 (4)	
H14	0.811 (3)	0.6565 (10)	0.574 (2)	0.046 (6)*	
C15	0.7118 (3)	0.74005 (9)	0.6470 (2)	0.0391 (5)	
H15	0.808 (3)	0.7634 (10)	0.719 (3)	0.057 (6)*	

### Atomic displacement parameters (Å^2^)

**Table:**

	*U*^11^	*U*^22^	*U*^33^	*U*^12^	*U*^13^	*U*^23^
C1	0.0225 (7)	0.0236 (9)	0.0259 (8)	0.0002 (7)	0.0035 (6)	0.0003 (7)
O1	0.0505 (7)	0.0247 (7)	0.0282 (6)	0.0088 (6)	0.0053 (5)	0.0019 (5)
C2	0.0274 (8)	0.0263 (9)	0.0219 (8)	−0.0003 (7)	0.0038 (6)	0.0000 (7)
N2	0.0501 (9)	0.0318 (9)	0.0252 (7)	0.0080 (7)	0.0062 (7)	−0.0004 (7)
O21	0.1138 (13)	0.0717 (12)	0.0334 (7)	0.0473 (10)	0.0244 (8)	0.0002 (8)
O22	0.1215 (14)	0.0427 (9)	0.0296 (7)	0.0315 (9)	0.0057 (8)	0.0062 (7)
C3	0.0266 (8)	0.0239 (9)	0.0299 (9)	0.0008 (7)	0.0070 (7)	−0.0040 (8)
C4	0.0280 (8)	0.0209 (9)	0.0321 (9)	0.0018 (7)	0.0077 (7)	0.0050 (7)
N4	0.0378 (8)	0.0286 (9)	0.0385 (8)	0.0024 (7)	0.0084 (7)	0.0083 (7)
O41	0.1011 (12)	0.0457 (9)	0.0393 (8)	0.0197 (8)	0.0173 (8)	0.0183 (7)
O42	0.0632 (9)	0.0264 (7)	0.0563 (9)	0.0118 (6)	0.0165 (7)	0.0024 (7)
C5	0.0292 (8)	0.0304 (10)	0.0223 (8)	−0.0002 (7)	0.0052 (7)	0.0037 (8)
C6	0.0261 (8)	0.0239 (9)	0.0250 (8)	−0.0007 (7)	0.0067 (6)	−0.0028 (7)
N6	0.0395 (8)	0.0282 (8)	0.0265 (7)	−0.0004 (7)	0.0093 (6)	−0.0008 (6)
O61	0.0498 (8)	0.0378 (8)	0.0451 (8)	0.0136 (7)	0.0134 (6)	−0.0063 (6)
O62	0.0563 (8)	0.0575 (10)	0.0419 (8)	−0.0018 (7)	−0.0060 (7)	−0.0211 (7)
N11	0.0479 (9)	0.0240 (8)	0.0329 (8)	−0.0010 (8)	0.0044 (7)	−0.0042 (7)
C12	0.0424 (10)	0.0252 (9)	0.0256 (8)	−0.0034 (8)	0.0067 (7)	−0.0002 (7)
C12A	0.0426 (12)	0.0519 (15)	0.0477 (12)	−0.0025 (11)	0.0000 (10)	0.0012 (12)
N13	0.0501 (9)	0.0230 (8)	0.0293 (8)	−0.0043 (7)	0.0077 (7)	−0.0044 (7)
C14	0.0426 (11)	0.0329 (11)	0.0381 (10)	0.0036 (9)	0.0084 (9)	0.0046 (9)
C15	0.0416 (10)	0.0374 (12)	0.0356 (10)	−0.0071 (9)	−0.0005 (8)	0.0006 (9)

### Geometric parameters (Å, °)

**Table:**

C1—O1	1.2557 (19)	N6—O61	1.2193 (18)
C1—C2	1.441 (2)	N6—O62	1.2226 (19)
C1—C6	1.450 (2)	N11—C12	1.329 (2)
C2—C3	1.376 (2)	N11—C15	1.375 (2)
C2—N2	1.459 (2)	N11—H11	0.89 (2)
N2—O22	1.2067 (19)	C12—N13	1.319 (2)
N2—O21	1.2145 (19)	C12—C12A	1.472 (3)
C3—C4	1.377 (2)	C12A—H12A	0.91 (3)
C3—H3	0.918 (18)	C12A—H12B	0.96 (3)
C4—C5	1.389 (2)	C12A—H12C	0.87 (3)
C4—N4	1.444 (2)	N13—C14	1.370 (2)
N4—O42	1.2200 (19)	N13—H13	0.86 (2)
N4—O41	1.2245 (19)	C14—C15	1.336 (3)
C5—C6	1.365 (2)	C14—H14	0.92 (2)
C5—H5	0.911 (19)	C15—H15	0.96 (2)
C6—N6	1.457 (2)		
			
O1—C1—C2	125.83 (14)	O61—N6—O62	123.62 (15)
O1—C1—C6	122.88 (14)	O61—N6—C6	118.85 (14)
C2—C1—C6	111.17 (14)	O62—N6—C6	117.53 (14)
C3—C2—C1	124.06 (14)	C12—N11—C15	109.23 (16)
C3—C2—N2	115.19 (14)	C12—N11—H11	123.3 (13)
C1—C2—N2	120.72 (14)	C15—N11—H11	126.7 (13)
O22—N2—O21	121.74 (15)	N13—C12—N11	107.04 (16)
O22—N2—C2	120.29 (14)	N13—C12—C12A	126.31 (18)
O21—N2—C2	117.97 (15)	N11—C12—C12A	126.64 (18)
C2—C3—C4	119.73 (16)	C12—C12A—H12A	112 (2)
C2—C3—H3	120.5 (11)	C12—C12A—H12B	112.4 (16)
C4—C3—H3	119.7 (11)	H12A—C12A—H12B	110 (2)
C3—C4—C5	121.00 (15)	C12—C12A—H12C	111 (2)
C3—C4—N4	118.57 (15)	H12A—C12A—H12C	104 (3)
C5—C4—N4	120.34 (15)	H12B—C12A—H12C	107 (3)
O42—N4—O41	122.90 (16)	C12—N13—C14	110.12 (16)
O42—N4—C4	118.99 (15)	C12—N13—H13	125.4 (15)
O41—N4—C4	118.10 (15)	C14—N13—H13	124.5 (15)
C6—C5—C4	118.21 (15)	C15—C14—N13	106.55 (17)
C6—C5—H5	120.2 (12)	C15—C14—H14	132.4 (13)
C4—C5—H5	121.6 (12)	N13—C14—H14	121.0 (13)
C5—C6—C1	125.60 (15)	C14—C15—N11	107.06 (17)
C5—C6—N6	116.91 (14)	C14—C15—H15	132.4 (13)
C1—C6—N6	117.40 (14)	N11—C15—H15	120.5 (13)
			
O1—C1—C2—C3	179.72 (16)	C4—C5—C6—C1	−0.5 (2)
C6—C1—C2—C3	−4.2 (2)	C4—C5—C6—N6	175.84 (13)
O1—C1—C2—N2	−2.4 (2)	O1—C1—C6—C5	−179.73 (16)
C6—C1—C2—N2	173.62 (14)	C2—C1—C6—C5	4.1 (2)
C3—C2—N2—O22	−163.80 (17)	O1—C1—C6—N6	3.9 (2)
C1—C2—N2—O22	18.2 (2)	C2—C1—C6—N6	−172.26 (13)
C3—C2—N2—O21	16.4 (2)	C5—C6—N6—O61	129.43 (16)
C1—C2—N2—O21	−161.61 (17)	C1—C6—N6—O61	−53.90 (19)
C1—C2—C3—C4	0.9 (2)	C5—C6—N6—O62	−51.0 (2)
N2—C2—C3—C4	−177.07 (14)	C1—C6—N6—O62	125.64 (17)
C2—C3—C4—C5	3.3 (2)	C15—N11—C12—N13	−0.36 (19)
C2—C3—C4—N4	179.90 (14)	C15—N11—C12—C12A	178.83 (19)
C3—C4—N4—O42	−6.2 (2)	N11—C12—N13—C14	0.53 (19)
C5—C4—N4—O42	170.49 (15)	C12A—C12—N13—C14	−178.67 (18)
C3—C4—N4—O41	174.92 (16)	C12—N13—C14—C15	−0.49 (19)
C5—C4—N4—O41	−8.4 (2)	N13—C14—C15—N11	0.25 (19)
C3—C4—C5—C6	−3.4 (2)	C12—N11—C15—C14	0.1 (2)
N4—C4—C5—C6	−179.99 (15)		

### Hydrogen-bond geometry (Å, °)

**Table:**

*D*—H···*A*	*D*—H	H···*A*	*D*···*A*	*D*—H···*A*
C5—H5···O21^i^	0.911 (19)	2.478 (19)	3.378 (2)	169.4 (16)
N11—H11···O1	0.89 (2)	1.95 (2)	2.8357 (19)	172.9 (19)
N13—H13···O1^ii^	0.86 (2)	2.09 (2)	2.819 (2)	143 (2)
N13—H13···O22^ii^	0.86 (2)	2.14 (2)	2.782 (2)	131.3 (19)

Symmetry codes: (i) *x*, *y*, *z*−1; (ii) *x*, −*y*+3/2, *z*−1/2.
